# Precarious transition: a mortality study of South African ex-miners

**DOI:** 10.1186/s12889-018-5749-2

**Published:** 2018-07-11

**Authors:** Kim Bloch, Leigh F. Johnson, Mlindeli Nkosi, Rodney Ehrlich

**Affiliations:** 1 Centre for Environmental and Occupational Health Research, School of Public Health and Family Medicine, University of Cape Town, Observatory 7925, Cape Town, South Africa; 2 Centre for Infectious Disease Epidemiology and Research, School of Public Health and Family Medicine, University of Cape Town, Cape Town, South Africa; 3 Actuarial Science Department, University of Cape Town, Cape Town, South Africa

**Keywords:** Mining, South Africa, Mortality, Silicosis, HIV, Tuberculosis

## Abstract

**Background:**

Despite their burden of a triple epidemic of silicosis, tuberculosis and HIV infection, little is known about the mortality experience of miners from the South African mining industry once they leave employment. Such information is important because of the size and dispersion of this population across a number of countries and the progressive nature of these diseases.

**Methods:**

This study included 306,297 South African miners who left the industry during 2001–2013. The study aimed to calculate crude and standardised mortality rates, identify secular trends in mortality and model demographic and occupational risk factors for mortality.

**Results:**

Crude mortality rates peaked in the first year after exit (32.8/1000 person-years), decreasing with each year from exit. Overall mortality was 20% higher than in the general population. Adjusted annual mortality halved over the 12 year period. Mortality predictors were being a black miner [adjusted hazard ratio (aHR) 3.30; 95% confidence interval (CI) 3.15–3.46]; underground work (aHR 1.33; 95% CI 1.28–1.39); and gold aHR 1.15 (95% CI 1.12–1.19) or multiple commodity employment (aHR 1.15; 95% CI 1.11–1.19).

**Conclusions:**

This is the first long-term mortality assessment in the large ex-miner population from the South African mining industry. Mortality patterns follow that of the national HIV-tuberculosis epidemic and antiretroviral treatment availability. However, ex-miners have further elevated mortality and a very high mortality risk in the year after leaving the workforce. Coordinated action between the mining industry, governments and non-governmental organisations is needed to reduce the impact of this precarious transition.

**Electronic supplementary material:**

The online version of this article (10.1186/s12889-018-5749-2) contains supplementary material, which is available to authorized users.

## Background

It has been belatedly recognised by the Southern African and international communities that the triple disease burden of silicosis, HIV infection and tuberculosis among the very large population of miners and ex-miners in Southern Africa constitutes a public health disaster [1, 2]. Drivers of the co-epidemic include industry related factors such as silica dust inhalation, transmission in congregate settings, and migrancy [3–5]. In response, the Global Fund to Fight AIDS, Tuberculosis and Malaria, the World Health Organization, the World Bank and the National Tuberculosis Programmes of Southern African countries have launched an ambitious programme to combat tuberculosis in mining populations in the Southern African region [6]. While this initiative aims to strengthen tuberculosis control programmes in affected countries, a specific goal is the tracing, screening and treatment of former miners.

The South African mining industry has been, and continues to be, reliant on migrant labour from rural areas of South Africa and neighbouring Southern African countries. Between 1973 and 2012, 1.64 million miners, mostly internal or foreign migrants, were recruited through the primary regional recruitment agency, TEBA Ltd. (“TEBA”), of whom 27% were from countries outside South Africa [7].

Given that many miners leave the industry for ill-health reasons and that ex-miners generally lose their access to company medical services, excess mortality would be expected among ex-miners relative to both working miners and the general population. This would be particularly so for the gold mining industry, where silica exposure is concentrated, and which accordingly suffers higher prevalences of tuberculosis and silicosis than the other major commodity sectors of platinum group metals (“platinum”) and coal [8–10].

While active gold miners have been the subject of a number of studies of morbidity and mortality, particularly from tuberculosis, silicosis, and HIV, relatively little is known about miners who have left the industry. Exceptions are three cross-sectional surveys of former gold miners, from the Transkei region of South Africa [11], Botswana [12], and Lesotho [13], which revealed a high prevalence of silicosis, tuberculosis, chronic obstructive pulmonary disease and HIV infection. Of these studies, the Transkei and Botswana studies were heavily weighted towards miners who were older, had longer service duration and/or had left the industry a number of years previously, and were therefore survivor groups. Only the Lesotho study was able to estimate mortality shortly after exit from the industry, revealing a very high 1 year mortality rate of 29 per 1000 [14].

The purpose of this study was to provide mortality information on this key ex-miner population. The analysis made use of data from the TEBA database and the South African death register - the National Population Register. The first objective was to calculate mortality rates among South African miners who left the mining industry between 2001 and 2013 and to compare these to rates in the general population. The second objective was to investigate trends in mortality over this period and the third was to identify demographic and occupational factors associated with mortality.

## Methods

### Selection of participants

The TEBA database used for this study covered the period 1973 to 2013 and includes 950,844 South African current and former miners from all commodity sectors, with their demographic and occupational information such as length of service, type of commodity, occupational category and nature of employment contract. Table 1 lists these variables and their definitions. No health, behavioural or cause of death information is available on the TEBA database. Also, while gold and platinum recruitment is believed to be reasonably well represented on the TEBA database during this period, recruitment into the coal industry is underrepresented [7].

**Table 1:** Explanation of variables from the database used for this study

Variable on database	Comment or further description	Derived Variables
South African ID number	Available only to statistician with access to South African Rapid Mortality Surveillance and not to researchers.	Vital status and date of death if deceased, 2011–2013.
Unique key	TEBA randomly assigned number available to the researchers.	
Racial ascription (race)^a^	Four category classification formalised under apartheid, and in continuing usage for most official purposes – “Asian, black, coloured, white”.	
Contract days	Sum of all days worked across all contracts.	Cumulative employment across all contracts.
Commodity mined	Gold, platinum group metals, coal, “other” (antimony, asbestos, chrome, copper, diamond, granite, iron, lead, lime, tin, vanadium).^b^	Commodity sector exclusively worked. Mixed service across sectors.
Occupational category	Underground, surface.	Occupational category exclusively worked. Mixed employment across occupational categories.
Nature of employment contract	Miners employed by the mining company distinguished from those employed by a third party contractor or via labour brokers.	

^a^Black miners, the majority of whom are or were migrants recruited from rural areas of South Africa and neighbouring countries, make up most of the mining workforce, and whites a significant minority. These categories have strong economic and social correlates, including in this context, occupational grade, earnings, area of origin, and lifetime health experience

^b^Includes unassigned employees recorded as “Medical”, “Security”, “Training” and “Sundry”

Vital status of the former miners was obtained by linking South African Identification Numbers (IDs) recorded in the TEBA database to the National Population Register. This linkage was carried out by the South African Medical Research Council in October 2013, drawing on death data acquired from the South African Department of Home Affairs as part of an ongoing Rapid Mortality Surveillance programme [15]. This programme does not include cause of death information. The TEBA file with personal IDs and vital status was returned by the Medical Research Council to TEBA. The researchers received only an anonymised file of the linked database from TEBA to maintain confidentiality.

Three levels of exclusion criteria were applied to the TEBA file of 950,844 miners of South African nationality. Since the National Population Register was regarded as less accurate prior to 2001, individuals who left the mining industry prior to 1 September, 2001, were excluded, which reduced the number of records to 335,421 individuals. In addition, individuals who died on or before their date of leaving the workforce were excluded, which further reduced the dataset to 309,645 individuals. Lastly, all individuals who left the workforce after 30 September, 2013, the censoring date, were excluded, resulting in the final study size of 306,297 South African former miners.

### Statistical analysis

All statistical analyses were performed using STATA 13.0 statistical software. Standardised mortality ratios (SMRs) were calculated by dividing the number of observed deaths by the number of deaths that would be expected in a sample of the general South African population with the same age, gender and racial ascription (“race”) distribution and the same length of follow-up by calendar year. The Actuarial Society of South Africa 2008 full model (ASSA2008) was used to estimate expected mortality rates amongst the general population by age, gender, racial ascription and calendar year [16].

Survival time was calculated as the total number of days between the date of termination of employment in the mining industry and the earlier of the two dates - the date of death or the date of censoring, 30 September, 2013. A Cox proportional hazards model was used to identify demographic and occupational predictors of mortality while adjusting for covariates.

Schoenfeld residuals were used to test the proportionality assumption. Owing to non-proportionality, additional sensitivity analyses were performed that compared follow-up restricted to the first 2 years after exit to follow-up restricted to durations longer than 2 years after exit.

## Results

### Demographic and occupational characteristics

Demographic and occupational characteristics of the 306,297 study participants are presented in Table 2. The majority of miners were black men (79.5%) and the median age at exit 35.9 years. Over two thirds (69.3%) had worked in one sector exclusively – predominantly in gold (42.5%). A total of 19.8% of miners had worked across multiple sectors, of whom 82.6% had worked at some time in gold mining. Almost 80% of miners had worked underground exclusively or both underground and in a surface job. The large majority (81%) had spent under 10 years in employment in the industry with median years of service 1.3 in gold, 1.0 in platinum and 3.8 in coal respectively. Median service length was much longer in those who had worked in multiple commodity sectors (5.8 years) or in both underground and surface jobs (9.6 years).

**Table 2:** Demographic and occupational characteristics of South African ex-miners who left the industry 2001–2013, with crude mortality rates (*N* = 306,297)

Characteristic		*N*	%	Crude mortality rate (per 1000 person-yrs)
TOTAL		306,297	100	23.1
Gender				
Male		290,765	94.9	23.6
Female		15,532	5.1	10.5
Racial ascription				
Black		255,364	83.4	26.4
White		46,857	15.3	8.0
Coloured		3453	1.1	13.2
Indian		507	0.2	8.0
Unknown^a^		116	3.8	5.2
Commodity				
Gold only		130,037	42.5	25.3
Platinum only		74,507	24.3	19.8
Coal only		3588	1.2	6.2
Other only		4080	1.3	9.5
Multiple sectors^b^		60,551	19.8	32.1
Unknown		33,534	11.0	11.9
Occupational category				
Underground only		155,093	50.6	21.5
Surface only		64,582	21.1	15.5
Underground and surface		80,412	26.3	34.3
Unknown		6210	2.0	5.8
Age at exit (years)				
15–24.9		41,704	13.6	8.8
25–34.9		103,557	33.8	18.7
35–44.9		75,611	24.7	30.6
45–54.9		59,024	19.3	29.3
55–64.9		25,624	8.4	25.6
≥ 65		777	0.3	23.5
Time in employment (years)				
0–4.9		217,860	71.1	18.3
5–9.9		33,173	10.8	33.3
10–14.9		29,861	9.7	37.9
15–19.9		16,063	5.2	34.3
20–24.9		5913	1.9	24.1
≥ 25		3427	1.1	22.3
Calendar year of exit (years)				
2001–2004		66,437	21.7	25.0
2005–2009		117,358	38.3	22.9
2010–2013		122,502	40.0	17.8

^a^“Unknown” category comprises missing, null or negative data. ^b^ “Multiple sectors” includes any combination of more than one sector, including individuals who spent a period of time in a sector reported as ‘unknown’

The TEBA database included information on nature of employment contract, i.e. whether employment was by the mining company or via a third party (“contractor”). The latter group merits particular attention because of their increased vulnerability [7, 17]. However, exploratory analysis of the distribution between company employees and contractors suggested considerable misclassification or other error. This variable was thus not analysed further.

### Crude mortality rates

A total of 33,097 deaths were observed over 1,433,700 person-years, giving an overall crude mortality rate over the approximately 12 year period of 23.1 per 1000 person-years (Table 2). Overall crude mortality rates were highest among those with mixed employment (32.1 per 1000 person-years), followed by exclusive gold (25.3 per 1000 person-years), exclusive platinum (19.8 per 1000 person-years and exclusive coal (6.2 per 1000 person-years).

### Standardised mortality ratios

Table 3 reports SMRs of the study population relative to the general South African population, standardizing on gender, racial ascription, age and calendar year. The overall SMR was 1.20, i.e. this ex-miner sample had a mortality rate 20% higher than that of the general population. SMRs of 1.18 and 1.20 were also recorded for black ex-miners and male ex-miners respectively relative to the equivalent strata in the general population. There was no excess mortality among female ex-miners. Of note is that white ex-miners had a 62% excess mortality relative to the white general population. When stratified by 5-year age groups, the largest disparity in mortality between ex-miners and the general population was in the youngest stratum, 20–24.9 years, with such ex-miners experiencing a 79% higher mortality rate than the general population of the same age.

**Table 3:** Standardised mortality ratios (SMR) of South African ex-miners who left the industry 2001–2013, relative to the general South African population (*N* = 306,297)

Characteristic	Actual deaths	Expected deaths	Adjusted SMR^a^	95% confidence interval
Racial ascription				
Asian	20	21	0.97	0.54–1.39
Black	30,918	26,187	1.18	1.17–1.19
Coloured	221	211	1.05	0.91–1.18
White	1935	1195	1.62	1.55–1.69
Gender				
Male	32,492	27,016	1.20	1.19–1.22
Female	602	599	1.01	0.93–1.09
Current age (years)				
20–24.9	595	332	1.79	1.65–1.93
25–29.9	2443	1771	1.38	1.32–1.43
30–34.9	4338	3245	1.34	1.30–1.38
35–39.9	5276	3602	1.46	1.43–1.50
40–44.9	5504	3732	1.47	1.44–1.51
45–49.9	5378	3977	1.35	1.32–1.39
50–54.9	4310	3766	1.14	1.11–1.18
55–59.9	2761	3126	0.88	0.85–0.92
60–64.9	1676	2422	0.69	0.66–0.73
65–69.9	645	1207	0.53	0.49–0.58
≥ 70	154	430	0.36	0.30–0.41
Total	33,094^b^	27,614	1.20	1.19–1.21

^a^SMRs controlled for calendar year, racial ascription, gender and age

^b^3 deaths from the study population excluded as racial ascription “unknown”

### Trends in mortality

**Fig. 1:**
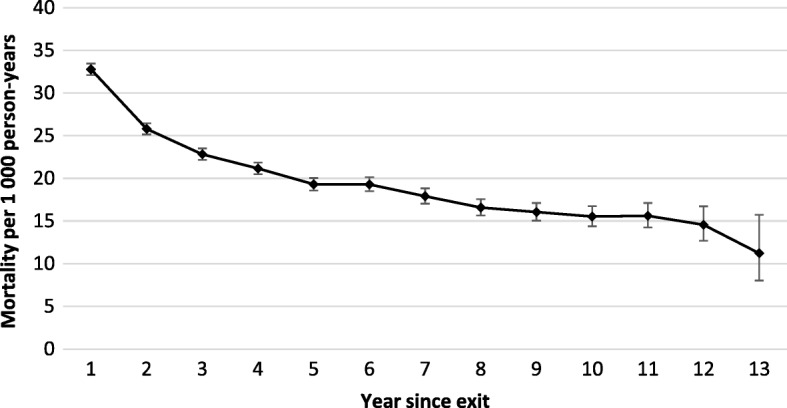
Crude mortality rate among South African miners by year since exit from the industry, 2001–2013 (*n* = 306,297). Each point on the curve represents the mortality rate over the preceding year, e.g. year 2 refers to the rate over the period 1.0–1.9 years after exit. Year 13 is an incomplete year. See Additional file 1: Table S1

Figure 1 charts the trend in mortality rates by years since exit from the workforce over the period 2001–2013. [(Additional file 1: Table S1) reports the data on which Fig. 1 is based]. During this period, mortality rates peaked in the first year after leaving employment at 32.8 per 1000 person years and decreased continuously and significantly as the year from exit increased (overall rate ratio 0.93 for every 1 year increase in duration from exit, 95% CI 0.92–0.93).

Table 4 presents the results of the crude and adjusted Cox proportional hazards models. After controlling for all other variables, there was a significant mortality rate decline in the later calendar periods of workforce exit, with the mortality rate among ex-miners who exited in the period 2010–2013 being less than half that of ex-miners who exited in the period 2001–2004.

**Table 4:** Occupational and demographic predictors of mortality in South African ex-miners who left the industry 2001–2013 (*N* = 306,297)^a^

Characteristic	Unadjusted hazard Ratio	Adjusted hazard ratio	95% confidence interval (adjusted)
Gender			
Female	1.00	1.00	–
Male	2.36	1.57	1.45–1.71
Racial ascription			
White	1.00	1.00	–
Black	3.25	3.30	3.15–3.46
Coloured	1.64	1.84	1.60–2.12
Indian	1.00	1.33	0.86–2.07
Unknown	0.65	0.30	0.04–2.10
Age at exit (years)			
15–24.9	1.00	1.00	–
25–34.9	2.12	1.91	1.81–2.01
35–44.9	3.54	2.77	2.63–2.92
45–54.9	3.39	2.98	2.82–3.15
55–64.9	2.94	2.85	2.68–3.03
≥ 65	2.71	3.08	2.48–3.84
Calendar year of exit			
2001–2004	1.00	1.00	–
2005–2009	0.75	0.77	0.75–0.79
2010–2013	0.44	0.45	0.43–0.46
Occupational category			
Surface only	1.00	1.00	–
Underground only	1.37	1.21	1.17–1.25
Underground and surface	2.24	1.33	1.28–1.39
Commodity			
Platinum only	1.00	1.00	–
Gold only	1.35	1.15	1.12–1.19
Coal only	0.35	0.42	0.36–0.49
Multiple sectors	1.65	1.15	1.11–1.19
Other only	0.49	0.66	0.56–0.77
Unknown only	0.63	0.83	0.79–0.88
Time in employment (years)			
0–4.9	1.00	1.00	–
5–9.9	1.83	1.24	1.20–1.29
10–14.9	2.14	1.20	1.16–1.24
15–19.9	1.95	1.06	1.02–1.11
20–24.9	1.35	0.83	0.76–0.89
≥ 25	1.25	0.83	0.74–0.92

^a^Cox Proportional Hazards model: adjustment for all variables in table

### Demographic and occupational predictors of mortality

Table 4 shows also that after adjustment for all covariates, gender, racial ascription, commodity and occupational category were significant predictive factors of mortality: males relative to females [adjusted hazard ratio (aHR) 1.57]; black relative to white ex-miners (aHR 3.30); those exposed to underground work, or both underground and surface work, relative to exclusively surface work (aHR 121 and 1.33 respectively). Relative to former exclusive platinum miners, elevated mortality risk was evident among those with exclusive gold (aHR 1.15) or multiple commodity sector experience (aHR 1.15). Ex-coal miners had a substantially lower risk of mortality than ex-platinum miners (aHR 0.42) and by extension, than ex-gold miners. Duration of employment showed a complex association – ex-miners with 5–20 years of employment had significantly higher mortality rates after leaving the workforce than those with < 5 years and ≥ 20 years’ duration. These findings persisted when the analysis was restricted to those who had worked exclusively in gold mining.

The global Schoenfeld proportionality test indicated that the overall model violated the proportional hazards assumption (*p* < 0.001). In the sensitivity analysis (Additional file 1: Table S2) there were a number of differences between relative mortality rates during the first 2 years from exit and those during the period greater than 2 years from exit. Specifically, in the first 2 years mortality rates were highest in the 35–44 years age group, while at longer duration from exit mortality was highest in the age groups > 45 years. Miners who had worked underground (versus surface) were at a significantly increased relative mortality risk in the first 2 years after leaving the workforce, with this effect attenuated at longer durations from exit. Similarly, the relative increase in mortality of the group with 5–20 years of employment was not observable at longer duration from exit (Additional file 1: Table S2).

## Discussion

This study of over 300,000 ex-miners who left the industry between 2001 and 2013 is to our knowledge the first to estimate long-term mortality rates in the ex-miner population from the South African mining industry and the temporal, demographic and occupational influences on the risk of dying. We believe it is important information for the large-scale interventions currently being set up in Southern Africa to bring treatment services to mining populations [6], as well as to maintain the pressure for disease prevention and just compensation and family benefits. A number of questions are considered below.

### Are the findings consistent with what has been reported in other studies?

This study found a crude annual mortality rate of 23 per 1000 over the 12 year period, reaching 32.1 per 1000 person-years among those with mixed commodity employment (of whom 80% had gold mining service) and 26.3 per 1000 person-years among exclusive gold miners. For comparison, a similar annual mortality rate of 29 per 1000 was recorded in the study of ex-gold miners from Lesotho conducted between 1999 and 2001 within 2 years of exit [13]. Of the 18 decedents (age range 43–59 years) in the Lesotho study, 14 were HIV positive and 12 were recorded as having respiratory problems prior to their deaths.

More detailed information exists on miners tracked while in service. A study of a gold miner cohort between 1993 and 2002 recorded a very rapid rise in mortality rates in parallel with a rising HIV infection prevalence over this decade, with mortality reaching 91.9 per 1000 person-years in HIV positive miners and 18.5 per 1000 person-years in HIV negative miners by the period 2000–2002 [18]. Using an assumed 20% HIV prevalence (discussed below) in our study population, a weighted average of these mortality rates would yield a crude rate of 33 per 1000 person-years, again consistent with our findings for gold or mixed commodity miners.

A later study of South African platinum miners measured a peak crude annual death rate of 21.1 per 1000 miners in 2003, which included deaths after leaving the company that were ascertained from provident fund, hospital and statutory autopsy services [19]. Allowing for the fact that this would include miners with mixed gold and platinum experience, their rate is consonant with our crude rate of 19.8 per 1000 person-years in exclusive platinum miners. Fifty percent of deaths in their cohort were attributable to HIV related causes of which tuberculosis (21% of total) was the largest component. Another 22% of deaths were due to violence and injuries. Of note is that the mortality rates found in the above studies are much greater than the annual estimate of 10 deaths per 1000 gold miners over the decade prior to 2014 cited from mining industry sources [20], suggesting significant administrative underestimation of deaths.

### Are former miners at increased risk of dying compared to the general population?

The expectation was that migrant ex-miners would be at greater risk of mortality than the general population because of their triple burden of silicosis, tuberculosis and HIV infection and the contribution of ill-health to exit from the industry. This was confirmed by the excess mortality of 20%, both overall and among male black miners, relative to the general population.

Published estimates of HIV infection in mining populations date mainly from the early 2000s. The range across provinces from surveys conducted in 2000 and 2001 was 11.0 to 24.3% [21]. In the 2000/2001 study of Basotho former gold miners cited above, an HIV prevalence of 22.3% was found at baseline [13], while an HIV prevalence of 24.6% was recorded in 2002 at a large platinum mining company [22]. Data on HIV prevalence cross-classified by age, province and racial ascription are not available to calculate a standardized prevalence ratio for miners against the general population. Nevertheless, the above are high prevalences by any standard. HIV and tuberculosis are thus plausibly the main explanations for the high excess mortality in the ex-miner population, exacerbated by loss of access to mine medical services, at least in the larger companies, and to accessible state medical services in towns close to mining operations.

Of concern is that although their absolute numbers were relatively small, the excess mortality was greatest among the youngest ex-miners (20–25 years), declining by age group thereafter. This is an important finding and deserves further investigation. In the general population, HIV prevalence is much lower in this age stratum of men [23], but this may not be the case among young miners. Another consideration is the possible contribution of violence and injuries to mortality in young ex-miners, given that this cause made up one fifth of deaths in the platinum miner cohort cited above [19] and generally accounts for a high proportion of deaths among young men in South Africa [24].

Another notable finding was that white former miners, representing higher occupational and managerial levels in in the industry, had a 67% greater mortality than the comparable general population. This is higher than in the last study of this population of miners which covered the period 1970 to 1989 and which found an SMR of 129 [25]. The major contributors to excess mortality in that study were ischaemic heart disease, chronic obstructive pulmonary disease, lung cancer and liver cirrhosis. The authors noted very high smoking rates as major risk factors. The apparent secular increase in excess mortality among white miners leaving the industry also merits further investigation.

### Time trends

A key finding was the very high mortality in the first year after exit, at 32.8 per 1000 person-years, followed by a rapid and then slower decline over succeeding intervals from exit. Miners with 12 years from exit had less than half the mortality rate of those in their first year from exit. As argued earlier, the most likely explanation is a high proportion of HIV infected miners and/or miners with active tuberculosis leaving the industry because of ill-health and experiencing high early mortality. In the platinum cohort referred to earlier [26], mortality was highest in the first month of tuberculosis treatment. Risk factors for mortality in that study included HIV infection, older age, previous tuberculosis, and diagnostic uncertainty. Follow up autopsies on a subset of the cohort on tuberculosis treatment found a considerable discrepancy between clinically attributed and autopsy confirmed cause of death, more so in HIV positive miners. Pneumonia, pneumocystis pneumonia and other infections were the main non-tuberculosis causes found at autopsy.

In a separate publication on the same cohort [27], the authors described a “dramatic” increase in transfers out of the company tuberculosis programme with the growth of the HIV epidemic prior to the introduction of antiretroviral treatment. This loss of access to company medical services with their HIV and tuberculosis treatment programmes, particularly among miners returning to rural areas with poorer medical services, thus creates what can be termed a *precarious transition.* Industry sources have recently stated that 4 % of gold miners are medically boarded (“repatriated”) annually, mainly for lung disease [20]. The picture is thus one of a high risk of dying, and by implication serious morbidity if surviving, among miners in the transition period after leaving the industry.

Multivariate analysis revealed declining mortality by calendar year, with the mortality rate among those leaving the workforce in the period 2010–2013 being approximately half that among those leaving in the period 2001–2004. This is consistent with various lines of evidence noting a fall in adult mortality from around 2005, which is commonly attributed to the impact of antiretroviral treatment on HIV-related mortality [15, 28]. In the platinum cohort described earlier [19], mortality rose sharply between 1993 and 2002, declining thereafter, but with a second peak in 2009 that was not seen in the current study.

### Predictors of mortality

A question of interest was whether the contribution of cumulative silica exposure and silicosis to death in this population could be inferred, in the absence of morbidity or cause of death information, from differences in mortality between commodity sectors and underground versus surface employment. The combination of silicosis and HIV infection is known to be a potent risk factor for incident tuberculosis among gold miners [29] while miners with silicosis have been shown to have higher mortality rates while on tuberculosis treatment than miners without silicosis [30]. After adjustment for all covariates those who had worked exclusively in gold mining had a 15% greater mortality risk than miners who had worked exclusively in platinum. This comparison is of relevance because platinum mining carries a very low risk of silicosis [10]. Another indicator of silica dust exposure, underground relative to surface occupation, was associated with elevated mortality but only in the first 2 years after exit.

There was a relatively weak effect of employment duration on mortality in the strata with 5–20 years of employment relative to those with either < 5 years or ≥ 20 years, after adjusting for age. This was also observable only for the first 2 years after exit. These findings persisted when the analysis was restricted to miners who had worked in gold mining only.

There was thus a lack of effect on mortality of indicators of degree of exposure to silica. However, a silica effect cannot be ruled out. Only 81.2% of this ex-miner population had an employment duration of 10 years or less, a period within which silicosis is uncommon, whether detected radiologically [31] or at autopsy [32]. This sample therefore reflected a high turnover of relatively short service workers, a phenomenon likely to have been be accelerated by shrinkage of the gold mining industry as well as the growing engagement of short-service “contractors” [7]. The lack of an employment duration effect may reflect a healthy survivor phenomenon in which the distribution of those “leaving sick”, particularly due to HIV and TB, is weighted towards shorter service workers.

After controlling for occupational and personal factors, black miners had a mortality rate threefold that of white miners. This is consistent with higher rates of HIV and tuberculosis among black miners with a possible contribution from violence and injuries, as discussed earlier, and aggravated by reduced access of ex-miners to health services. This excess mortality is characteristic of a society in which socioeconomic status differences take on a strongly racialized form.

### Strengths and limitations

The completeness of death reporting is important to consider. While apartheid-era South Africa had a fragmented, non-standardised, non-inclusive registration system for births and deaths, the period of the study, 2001 to 2013, benefited from a greatly improved national death registration system [33]. During 1996–2001, approximately 84% of adult deaths were recorded by the vital registration system [34]. The proportion of adult deaths that were recorded increased to approximately 93% after 2001 as a result of intensified efforts of the post-apartheid government to maintain more accurate death records [35]. It is therefore unlikely that many deaths were missed.

The changing role of TEBA and of the representativeness of its database have been described in detail elsewhere [7]. In summary, the TEBA database is likely to represent a very high proportion of miners employed in the gold mining industry during this period, a substantial proportion of platinum miners and a minority of coal miners. Representativeness is likely to be lower for high employment grades than low employment grade workers, and accordingly higher for black than white employees. The mortality rates can therefore be generalized to black South African gold and platinum miners who left the industry during the period of the study. This is less confidently the case for coal miners and white employees.

We were not able to obtain cause of death data owing to the absence of these data in the National Population Register. Nor did we have access to morbidity information such as silicosis, HIV or tuberculosis status, or to information on risk factors for premature mortality such as smoking and alcohol intake. While the larger mines hold individual medical records through their company medical services, the TEBA database does not record this information. “Contractor” employment status data, although of great interest, were not considered reliable enough to use as a predictor of mortality. Finally, while the analysis was restricted to former miners of South African nationality, the consistency of the mortality rate with that found among ex-miners from Lesotho noted earlier [16] suggests that the picture among foreign national former miners is unlikely to be different. However, information about the fate of ex-miners from neighbouring countries remains sparse.

## Conclusions

There is now a substantial amount of tuberculosis and HIV morbidity and mortality information on workers on the South African gold mines, and to a lesser extent on the platinum mines. However, almost all of these findings are based on employed miners, with some limited tracking of cohorts to statutory autopsy or hospital admission post-exit. The current study attempted to extend our understanding of the fate of ex-miners, a substantial and growing population throughout Southern Africa. Our findings are consistent with the predominant influence of the lethal HIV-tuberculosis co-epidemic among mineworkers over the period studied and a reversal of this effect under the influence of the extension of antiretroviral treatment nationally.

The fate of ex-miners described in this report constitutes a spillover from occupational to public health in Southern Africa. Besides the impact on the miners themselves, the return of sick and dying migrants imposes resource and care burdens on families and communities that can least afford them [36]. The problem of loss of miners to tuberculosis treatment once they leave employment has been noted by mining medical personnel since effective tuberculosis treatment became available in the 1970s [37], but has only seriously entered the public health agenda during the last decade under the pressures of the HIV-TB co-epidemic and its additional treatment needs.

We make the following recommendations based on the results of this study and the conclusions we have drawn.

1. Detailed elements of a programme for coordinated management of sick miners returning home, focused on Lesotho, were published in 2008 [38]. These have been taken up at the regional level by an inter-ministerial Declaration [1] and through a protocol for regional harmonisation of tuberculosis treatment [39]. However, evidence of sustained implementation needs to be presented.
2. There is a need to strengthen the capacity of mining communities to understand the systemic factors underlying their predicament and to be able to advocate for needed services and compensation [40].
3. The data provided in this report suggest progress in reducing mortality, but such data need to be supplemented by ongoing health surveillance of the ex-miner population, including in neighbouring countries.
4. New programmes bringing large amounts or resources to tuberculosis treatment have an opportunity to link their activities to those of mining company medical services as well as regional health systems to ensure coverage of sick ex-miners in the precarious transition period described in this report.
5. The occupational and therefore preventable contribution of silica dust and silicosis to tuberculosis morbidity and mortality also needs to be recognised lest it disappear from view under the pressure of treatment targets.

## Additional file

Additional file 1: **Table S1.** Number of deaths, person years, and mortality rates of South Africa ex-miners by time since exit from mining service (2001–2013) (*N* = 306,297). **Table S2.** Cox model estimates stratified by durations 0–2 years and > 2 years after leaving workforce (*N* = 306,297). (DOCX 23 kb)

## Abbreviations

aHR: Adjusted hazard ratio
ASSA: Actuarial Society of South Africa
HIV: Human immunodeficiency virus
SMR: Standardised mortality ratio
TB: Tuberculosis
