# Supplementary material for: Precarious transition: a mortality study of South African ex-miners
Source: BMC Public Health. 2018 Jul 11;18:862. doi: 10.1186/s12889-018-5749-2 (PMC6042385; doi:10.1186/s12889-018-5749-2)
Supplement: Supplementary file 1 — Table S1. Number of deaths, person years, and mortality rates of South Africa ex-miners by time since exit from mining service (2001–2013) (N = 306,297). Table S2. Cox model estimates stratified by durations 0–2 years and > 2 years after leaving workforce (N = 306,297). (DOCX 23 kb) [file 12889_2018_5749_MOESM1_ESM.docx]

**ADDITIONAL FILE 1**

**Table S1.** Number of deaths, person years, and mortality rates of

South Africa ex-miners by time since exit from mining service (2001-2013)

(N= 306 297)^a^

| **Duration of time since exit (years)** | **No. of deaths** | **Person years (1 000s)** | **Mortality rate per 1 000 person-years** | **95% confidence interval** |
| --- | --- | --- | --- | --- |
| 0-0.9 | 9 123 | 278.3 | 32.8 | 32.1-33.5 |
| 1-1.9 | 6 010 | 232.9 | 25.8 | 25.2-26.5 |
| 2-2.9 | 4 526 | 198.3 | 22.8 | 22.2-23.5 |
| 3-3.9 | 3 630 | 171.5 | 21.2 | 20.5-21.9 |
| 4-4.9 | 2 715 | 140.7 | 19.3 | 18.6-20.0 |
| 5-5.9 | 2 118 | 109.8 | 19.3 | 18.5-20.1 |
| 6-6.9 | 1 521 | 84.9 | 17.9 | 17.0-18.8 |
| 7-7.9 | 1 177 | 71.0 | 16.6 | 15.7-17.6 |
| 8-8.9 | 922 | 57.4 | 16.0 | 15.0-17.1 |
| 9-9.9 | 665 | 42.8 | 15.5 | 14.4-16.8 |
| 10-10.9 | 455 | 29.1 | 15.6 | 14.2-17.1 |
| 11-11.9 | 201 | 13.8 | 14.6 | 12.7-16.7 |
| 12-12.9 | 34 | 3.0 | 11.2 | 8.0-15.7 |
| **Total** | **33 097** | **1 433.7** | **23.1** | **22.8-23.3** |

^a^Presented as Fig. 1.

**Table S2.** Cox model estimates stratified by durations 0-2 years and >2 years after leaving workforce (N= 306 297)

| **Covariate** | **First two years of follow-up** | | **Follow-up duration >2 years** | |
| --- | --- | --- | --- | --- |
|  | **Hazard ratio** | **95% confidence interval** | **Hazard ratio** | **95% confidence interval** |
| ***Gender*** |  |  |  |  |
| Female | 1.00 | - | 1.00 | - |
| Male | 1.48 | 1.32 - 1.66 | 1.63 | 1.45 - 1.83 |
| ***Racial ascription*** |  |  |  |  |
| White | 1.00 | - | 1.00 | - |
| Black | 3.40 | 3.16 - 3.66 | 3.24 | 3.05 - 3.44 |
| Coloured | 1.53 | 1.19 - 1.96 | 2.01 | 1.69 - 2.38 |
| ***Industry*** |  |  |  |  |
| Platinum only | 1.00 | - | 1.00 | - |
| Gold only | 1.11 | 1.06 - 1.16 | 1.17 | 1.12 - 1.22 |
| Coal only | 0.35 | 0.26 - 0.45 | 0.47 | 0.39 - 0.57 |
| Multiple sectors | 1.13 | 1.07 - 1.19 | 1.15 | 1.09 - 1.21 |
| ***Occupational risk category*** |  |  |  |  |
| Surface only | 1.00 | - | 1.00 | - |
| Underground only | 1.50 | 1.42 - 1.59 | 1.04 | 1.00 - 1.09 |
| Underground and surface | 1.64 | 1.54 - 1.75 | 1.18 | 1.12 - 1.24 |
| ***Age at leaving workforce (years)*** |  |  |  |  |
| 15-24.9 | 1.00 | - | 1.00 | - |
| 25-34.9 | 2.21 | 2.03 - 2.4 | 1.74 | 1.63 - 1.86 |
| 35-44.9 | 3.50 | 3.21 - 3.81 | 2.35 | 2.20 - 2.51 |
| 45-54.9 | 3.37 | 3.08 - 3.68 | 2.75 | 2.56 - 2.95 |
| 55-64.9 | 2.68 | 2.42 - 2.97 | 3.03 | 2.80 - 3.28 |
| ≥65 | 2.97 | 2.1 - 4.2 | 3.25 | 2.45 - 4.31 |
| ***Date of leaving service (years)*** |  |  |  |  |
| 2001-2004 | 1.00 | - | 1.00 | - |
| 2005-2009 | 0.83 | 0.8 - 0.86 | 0.73 | 0.70 - 0.75 |
| 2010-2013 | 0.46 | 0.44 - 0.48 | 0.52 | 0.48 - 0.57 |
| ***Total years in employment (years)*** |  |  |  |  |
| 0 - 4.9 | 1.00 | - | 1.00 | - |
| 5 - 9.9 | 1.46 | 1.38 - 1.53 | 1.06 | 1.00 - 1.11 |
| 10 – 14.9 | 1.44 | 1.36 - 1.51 | 1.04 | 0.99 - 1.09 |
| 15 – 19.9 | 1.25 | 1.17 - 1.34 | 0.94 | 0.89 – 1.00 |
| 20 -24.9 | 0.90 | 0.79 - 1.01 | 0.77 | 0.70 - 0.86 |
| ≥25 | 0.84 | 0.71 – 1.00 | 0.80 | 0.70 - 0.91 |
